# *Spiroplasma* affects host aphid proteomics feeding on two nutritional resources

**DOI:** 10.1038/s41598-018-20497-9

**Published:** 2018-02-06

**Authors:** Aline Sartori Guidolin, Thaís Regiani Cataldi, Carlos Alberto Labate, Frederic Francis, Fernando Luis Cônsoli

**Affiliations:** 1 Insect Interactions Laboratory, Department of Entomology and Acarology, ESALQ, University of São Paulo, Av. Pádua Dias 11, 13418-900 Piracicaba, São Paulo, Brazil; 2 Max Feffer Laboratory of Plant Genetics, Department of Genetics, ESALQ, University of São Paulo, Av. Pádua Dias 11, 13418-900 Piracicaba, São Paulo, Brazil; 3 Entomologie Fonctionnelle et Evolutive, Gembloux Agro-Bio Tech, Université de Liège, 2 Passage des Déportés, 5030 Gembloux, Belgium

## Abstract

Bacterial symbionts are broadly distributed among insects, influencing their bioecology to different degrees. Aphids carry a number of secondary symbionts that can influence aphid physiology and fitness attributes. *Spiroplasma* is seldom reported as an aphid symbiont, but a high level of infection has been observed in one population of the tropical aphid *Aphis citricidus*. We used sister isolines of *Spiroplasma*-infected (Ac-BS) and *Spiroplasma*-free (Ac-B) aphids reared on sweet orange (optimum host) and orange jasmine (suboptimum host) to demonstrate the effects of *Spiroplasma* infection in the aphid proteome profile. A higher number of proteins were differently abundant in aphids feeding on orange jasmine, indicating an impact of host plant quality. In both host plants, the majority of proteins affected by *Spiroplasma* infection were heat shock proteins, proteins linked to cell function and structure, and energy metabolism. *Spiroplasma* also induced changes in proteins involved in antimicrobial activity, carbohydrate processing and metabolism, amino acid synthesis and metabolism in aphids feeding on orange jasmine. We discuss on how the aphid host proteome is differentially affected by *Spiroplasma* infection when the host is exploiting host plants with different nutritional values.

## Introduction

All multicellular animals have symbiotic microbes that establish biochemical and physiological interactions with their hosts. In insects, microorganisms are known to participate in many of the host physiological processes, e.g. by influencing the host capacity to use host plants or in pathogen transmission efficiency as vector of plant diseases^1–3^. The combination of two metabolic systems (symbiont + host) has a variety of potential consequences to the host, such as in aphids that may carry a great diversity of them. Aphids are known to harbor their obligate symbiont *Buchnera aphidicola*^4,5^, and yet be associated with several facultative symbionts^6–10^.

*Buchnera aphidicola* and aphids have a long co-evolutionary history. They share several metabolic pathways related to amino acids synthesis, and *B. aphidicola* provides aphids with essential amino acids that are not produced by the host nor are acquired from phloem sap, its natural diet. In many instances, *B. aphidicola* uses precursors and enzymes from aphid metabolic pathways to contribute to aphid nutrition^3,11^. Facultative symbionts are diverse and the outcome of their interactions with their hosts are quite variable as they induce a plethora of phenotypes in host aphids^12^.

Secondary symbionts have also been related to host plant use in aphids, either by broadening or narrowing the insect host range^13,14^. However, the molecular and physiological mechanisms by which secondary symbionts interfere with host plant utilization and host range adaptation in aphids remains unclear. Proteomics is an interesting approach for the investigation and identification of metabolic processes and pathways differently expressed in an organism subjected to particular challenges. By example, proteomics of aphids saliva have been successfully applied to investigate aphid interactions with host plants and viruses aphids vector, as well as aphids response to stress conditions^15–19^. Aphid proteomics is also a growing area of research to investigate the effects of secondary symbionts that may have impact in the association with their hosts at different trophic levels^15–19^.

*Spiroplasma* is a common facultative symbiont reported in insects, but its role in host biology is still being questioned and explored^20^. *Spiroplasma* are known to be associated with at least 16 species of *Drosophila*, but the relationship of this symbiont with their *Drosophila* hosts may be quite variable^21–26^. In aphids, this symbiont was first shown in *Acyrthosiphon pisum* (Harris), and injections into “bacteria-free aphid lines” demonstrated that *Spiroplasma* negatively affected host biology^27^. Later, *Spiroplasma* was discovered to confer protection against a fungal entomopathogen due to aphid behavior manipulation. Aphids carrying *Spiroplasma* would drop off the plant and sporulate away from siblings, limiting the spread of the disease^28^.

However, *Spiroplasma* is not so frequently found in aphids as other secondary symbionts^29,30^. Nevertheless, previous work reported high infections rates of *Aphis citricidus* (Kirkaldy) and *Aphis aurantii*^31^. *Aphis citricidus* is an oligophagous species, restricted to *Citrus* as suitable host plants, although it may survive and reproduce poorly in orange jasmine (*Murraya paniculata*)^32^. Previous comparative biology studies of *A. citricidus* showed that sweet orange is an optimum host, while orange jasmine is a suboptimum host plant for aphid development^33^.

Thus, we aimed to investigate the changes induced by *Spiroplasma* infection in the proteome of *A. citricidus* and associated symbionts when feeding on sweet orange (optimum host plant) and on orange jasmine (suboptimum host plant) host plants. We report the overall effects of *Spiroplasma* infection on the proteomics of *A. citricidus* and discuss on its implications for host fitness and host plant use in this aphid.

## Results

The *Spiroplasma*-injected *A. citricidus* sister line was successfully infected as confirmed by diagnostic PCR (data not shown). Protein extraction and purification also yielded high quality protein samples with enough concentration for proteomic analyses.

**Figure 1:**
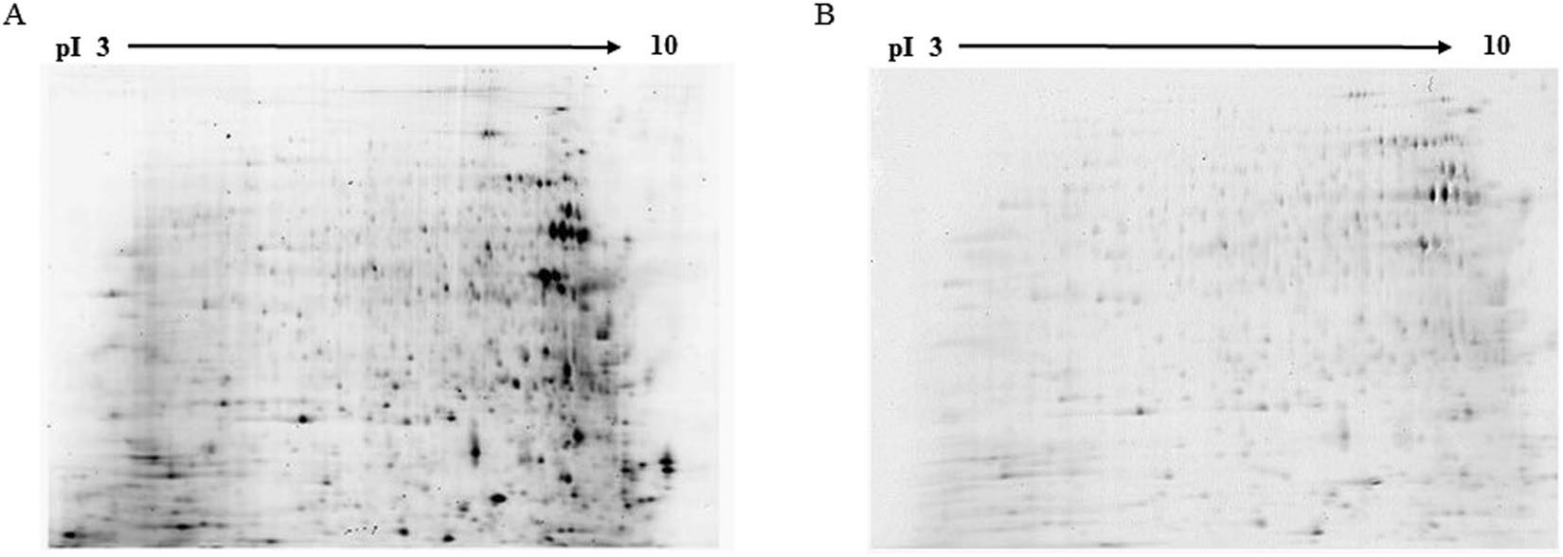
Representative 2D 12.5% SDS-PAGE gel of protein samples labeled with Cye3 from the Ac-B isoline reared on orange jasmine (**A**) and on sweet orange (**B**).

Gel images passed to all quality filters, and spots were evenly distributed (Fig. 1). Initially, 174 spots were differently abundant between *Spiroplasma* infected and *Spiroplasma* free lines, but only 92 had minimum sample volume for further analysis. Out of the 92 spots analyzed, 80 of them were identified. 53 out of these 80 spots were differently abundant between Ac-B and Ac-BS using orange jasmine as a host plant, while 27 differently abundant spots were identified in aphids reared on sweet orange as a host (Tables 1 and 2).

**Table 1:** Identification of differently abundant spots from *Spiroplasma*-free (Ac-B) and *Spiroplasma* infected (Ac-BS) isolines of *Aphis citricidus* feeding on sweet orange.

Spot	Normalized volume		ANOVA (*p-value*)	Fold change	Protein	Species	NCBInr gi or Uniprot entry name	Score	Mass	pI	N. peptides matched/searched	% coverage
	Control	*Spiroplasma*										
**Protein-Protein interactions**												
**Heat shock 60**												
531	1.013	1.308	0.007	1.3 (+)^*^	60 kDa chaperonin^2**^	*B. aphidicola Sg*	CH60_BUCAP	3615	57965		19/45	32.6
**Heat shock 70**												
193	0.792	0.990	0.008	1.3 (+)	uncharacterized protein^2^	*Acyrthosiphon pisum*	J9JWP2_ACYPI	4044	72182		26/48	39.3
194	0.952	1.094	0.05	1.1 (+)	uncharacterized protein^2^	*Acyrthosiphon pisum*	J9JWP2_ACYPI	3291	72182		27/48	40.4
203	0.984	1.140	<0.001	1.2 (+)	uncharacterized protein^2^	*Acyrthosiphon pisum*	J9JWD2_ACYPI	940	73920		13/65	24.1
204	0.915	1.076	0.01	1.2 (+)	chaperone protein DnaK^2^	*B. aphidicola Sg*	DNAK_BUCAP	1285	70401		14/68	32.4
214	0.988	1.094	0.014	1.1 (+)	heat shock protein cognate 3 precursor^1^	*Acyrthosiphon pisum*	gi|193716022	259	72993	5.2	28/45	47
215	0.819	1.082	0.009	1.3 (+)	uncharacterized protein^2^	*Acyrthosiphon pisum*	J9JJY7_ACYPI	1653	71670		15/49	22.6
217	0.948	1.130	0.03	1.2 (+)	uncharacterized protein^2^	*Acyrthosiphon pisum*	J9JJY7_ACYPI	2166	71670		17/49	31.9
262b	1.249	0.745	0.031	1.7 (−)	heat shock protein cognate 3 precursor^1^	*Acyrthosiphon pisum*	gi|193716022	95	72993	5.2	15/42	28
274	1.105	0.979	0.002	1.1 (−)	heat shock 70 kDa protein cognate 4^1^	*Acyrthosiphon pisum*	gi|193688192	169	72138	5.3	17/32	31
324	0.832	1.082	0.009	1.2 (+)	heat shock 70 kDa protein cognate 4-like^1^	*Acyrthosiphon pisum*	gi|193603578	150	71626	5.3	15/25	33
**Heat shock 90**												
131	0.875	1.074	0.03	1.2 (+)	uncharacterized protein^2^	*Acyrthosiphon pisum*	J9JP34_ACYPI	748	83759		10/69	16.2
**Calcium binding**												
883	1.308	0.977	0.005	1.3 (−)	ACYPI007505 protein^2^	*Acyrthosiphon pisum*	C4WYG9_ACYPI	747	17432		5/22	33.7
**Genetic transcription control**												
608	0.992	1.219	0.028	1.2 (−)	uncharacterized protein^2^	*Acyrthosiphon pisum*	J9JV14_ACYPI	2984	36544		17/28	55.8
**Energy metabolism**												
440	0.993	1.117	0.017	1.1 (+)	malyl-CoA lyase^1^	*Granulibacter bethesdensis*	gi|499950029	72	36167	5.8	6/14	21
788	1.327	1.078	0.022	1.2 (−)	uncharacterized protein^2^	*Acyrthosiphon pisum*	J9JQL8_ACYPI	1316	21268		4/17	23.0
801	0.812	0.952	0.02	1.2 (+)	glyceraldehyde-3-phosphate dehydrogenase^2^	*Acyrthosiphon pisum*	C4WYF8_ACYPI	521	35735		6/24	24.4
**Cell function and structure**												
363	0.878	1.025	0.01	1.2 (+)	tubulin beta-1 chain^2^	*Acyrthosiphon pisum*	D7RA98_ACYPI	4347	50558		28/29	54.4
610	1.1	1.25	0.025	1.1 (−)	uncharacterized protein^2^	*Acyrthosiphon pisum*	J9JLA2_ACYPI	3288	32571		9/27	25
637	1.2	1.214	0.047	1.2 (−)	ACYPI003527 protein^2^	*Acyrthosiphon pisum*	C4WTM0_ACYPI	215	22715		3/11	19.3
665	1.367	0.873	0.01	1.6 (−)	uncharacterized protein^2^	*Acyrthosiphon pisum*	X1XTT0_ACYPI	151	20386		2/8	12.8
893	0.927	1.08	<0.001	1.2 (+)	ACYPI005394 protein^2^	*Acyrthosiphon pisum*	C4WUV1_ACYPI	1374	20659		7/22	22.3
962	1.196	0.937	0.002	1.3 (−)	ACYPI008348 protein^2^	*Acyrthosiphon pisum*	C4WTD6_ACYPI	2421	11575		5/9	45.5
1038	1.1	0.968	0.019	1.1 (−)	putative cofilin/actin depolymerizing factor-like protein^2^	*Acyrthosiphon pisum*	Q1ZZP9_ACYPI	1411	17364		2/16	16.9
**Ion transport**												
262a	1.249	0.745	0.031	1.7 (−)	V-type proton ATPase catalytic subunit A^1^	*Acyrthosiphon pisum*	gi|193690627	116	68057	5	15/42	28
**Processing and degradation**												
713	0.883	1.040	0.009	1.2 (+)	ACYPI004563 protein^2^	*Acyrthosiphon pisum*	C4WYA2_ACYPI	413	24716		3/14	22.9
**Unknown**												
197	1.487	0.749	0.021	2.0 (−)	uncharacterized protein^2^	*Acyrthosiphon pisum*	J9K6K0_ACYPI	392	72647		9/33	14.6
825	1.032	1.153	0.018	1.1 (+)	ACYPI003440 protein^2^	*Acyrthosiphon pisum*	C4WUX5_ACYPI	1099	20238		3/20	18.6

^*^Protein abundance pattern in response to *Spiroplasma* infection. (+) = up-regulated protein, (−) = down-regulated protein.

^**^Number indicates the mass spectrometer used in the spot identification, 1 = Ultraflex II MALDI and 2 = *Synapt* G2 HDMS.

**Table 2:** Identification of differently abundant spots from *Spiroplasma*-free (Ac-B) and *Spiroplasma* infected (Ac-BS) isolines of *Aphis citricidus* feeding on orange jasmine.

Spot	Normalized volume		ANOVA (*p- value*)	Fold change	Protein	Species	NCBInr gi or Uniprot entry name	Score	Mass	pI	N. peptides matched/searched	% coverage
	control	*Spiroplasma*										
**Protein-Protein Interactions**												
**Heat shock 10**												
2318	0.509	1.664	<0.001	3.3 (+)*	10 kDa chaperonin^2**^	*B. aphidicola* 5 A	CH10_BUCA5	1703	10243		2/6	28.1
**Heat shock 60**												
776	1.651	0.759	0.004	2.0 (−)	60 kDa chaperonin^2^	*B. aphidicola Sg*	CH60_BUCAP	4001	57965		29/45	46.7
832	1.998	0.432	<0.001	4.6 (−)	60 kDa chaperonin^2^	*B. aphidicola Sg*	CH60_BUCAP	5376	57965		24/45	35.8
841	1.755	0.573	<0.001	3.1 (−)	60 kDa chaperonin^2^	*B. aphidicola Sg*	CH60_BUCAP	3742	57965		21/45	35.8
855	2.107	0.490	<0.001	4.3 (−)	60 kDa chaperonin^2^	*B. aphidicola Sg*	CH60_BUCAP	3200	57965		20/45	33.6
937	1.801	0.419	0.004	4.3 (−)	60 kDa chaperonin^2^	*B. aphidicola Sg*	CH60_BUCAP	463	57965		11/45	29.1
**Heat shock 70**												
417	1.344	0.472	0.002	2.8 (−)	heat shock 70 kDa protein 4^1^	*Acyrthosiphon pisum*	gi|193596448	59	89426	5.5	6/11	9
663	1.532	0.634	0.003	2.4 (−)	heat shock protein cognate 3 precursor^1^	*Acyrthosiphon pisum*	gi|193716022	257	72993	5.2	32/66	51
685	1.532	0.634	0.003	2.3 (−)	chaperone protein DnaK^2^	*B. aphidicola Sg*	DNAK_BUCAP	1404	70401		9/68	22.4
687	1.559	0.711	<0.001	2.2 (−)	uncharacterized protein^2^	*Acyrthosiphon pisum*	J9JJY7_ACYPI	2919	71671		19/49	33.8
693	1.453	0.529	<0.001	2.7 (−)	uncharacterized protein^2^	*Acyrthosiphon pisum*	J9JJY7_ACYPI	1246	71671		13/49	20.9
699a	1.543	0.641	0.007	2.4 (−)	heat shock 70 kDa protein cognate 4-like^1^	*Acyrthosiphon pisum*	gi|193603578	112	71626	5.3	16/45	31
699b	1.544	0.642	0.007	2.4 (−)	heat shock protein cognate 3 precursor^1^	*Acyrthosiphon pisum*	gi|193716022	112	72993	5.2	17/45	29
**Heat shock 90**												
397	1.298	0.529	<0.001	2.7 (−)	endoplasmin^1^	*Acyrthosiphon pisum*	gi|193643557	89	89250	4.8	14/35	16
524	0.579	2.168	0.014	3.7 (+)	uncharacterized protein^2^	*Acyrthosiphon pisum*	J9JP34_ACYPI	2221	83759		19/69	26.4
**Calcium binding**												
2128	0.717	1.600	<0.001	2.2 (+)	ACYPI000027 protein^2^	*Acyrthosiphon pisum*	Q1ZZQ3_ACYPI	1093	17649		3/13	22.1
**Energy metabolism**												
951	1.522	0.656	0.002	2.3 (−)	ATP synthase subunit alpha mitochondrial^1^	*Acyrthosiphon pisum*	gi|193666827	84	59986	9.1	12/39	22
971	1.600	0.614	0.001	2.6 (−)	uncharacterized protein^2^	*Acyrthosiphon pisum*	J9JNB2_ACYPI	199	48183		8/40	19.6
959	1.623	0.739	<0.001	2.2 (−)	ATP synthase subunit alpha^2^	*Acyrthosiphon pisum*	J9JYX3_ACYPI	1469	60023		11/50	21.6
1019	0.468	2.045	0.006	4.4 (+)	ATP synthase subunit alpha^2^	*Acyrthosiphon pisum*	J9JYX3_ACYPI	472	60023		7/50	13.2
1331	1.446	0.593	<0.001	2.4 (−)	malate dehydrogenase^2^	*Acyrthosiphon pisum*	Q201V2_ACYPI	1007	36103		9/23	28.9
1353	1.574	0.662	<0.001	2.4 (−)	glyceraldehyde-3-phosphate dehydrogenase^2^	*Acyrthosiphon pisum*	J9K9Z1_ACYPI	2993	35645		9/24	31.0
1760	0.709	1.631	<0.001	2.3 (+)	triosephosphate isomerase^2^	*Acyrthosiphon pisum*	C4WT45_ACYPI	293	27468		3/21	15.4
**Anti-microbial peptides**												
581	1.892	0.597	<0.001	3.2 (−)	repetitive proline-rich cell wall protein 2-like isoform X2^1^	*Acyrthosiphon pisum*	gi|641669847	111	72602	7.7	15/47	22
587	1.838	0.411	<0.001	4.5 (−)	repetitive proline-rich cell wall protein 2-like isoform X2^1^	*Acyrthosiphon pisum*	gi|641669847	82	72602	7.7	10/35	16
588	1.859	0.610	<0.001	3.0 (−)	repetitive proline-rich cell wall protein 2-like isoform X2^1^	*Acyrthosiphon pisum*	gi|641669847	94	72602	7.7	15/60	21
589	1.703	0.577	<0.001	2.9 (−)	repetitive proline-rich cell wall protein 2-like isoform X2^1^	*Acyrthosiphon pisum*	gi|641669847	115	72602	7.7	16/52	21
590	1.817	0.711	<0.001	3.0 (−)	repetitive proline-rich cell wall protein 2-like isoform X2^1^	*Acyrthosiphon pisum*	gi|641669847	113	72602	7.7	17/70	21
660	1.821	0.751	0.016	2.4 (−)	repetitive proline-rich cell wall protein 2-like isoform X2^1^	*Acyrthosiphon pisum*	gi|641669847	79	72602	7.7	8/20	13
**Cell function and structure**												
1264	0.641	1.474	<0.001	2.3 (+)	actin related protein 1^2^	*Acyrthosiphon pisum*	gi|217330650	141	42158	5.3	12/32	40
1265	0.411	1.897	<0.001	4.6 (+)	putative actin^2^	*Acyrthosiphon pisum*	Q201U9_ACYPI	175	42221		4/34	14.6
1356	0.460	1.591	<0.001	3.2 (+)	uncharacterized protein^2^	*Acyrthosiphon pisum*	C4WTU7_ACYPI	1192	32818		8/26	30.7
1541	0.606	1.584	<0.001	2.6 (+)	ACYPI003527 protein^2^	*Acyrthosiphon pisum*	C4WTM0_ACYPI	911	22715		6/11	40.4
1761	0.697	1.603	<0.001	2.3 (+)	ACYPI005885 protein^2^	*Acyrthosiphon pisum*	C4WYE4_ACYPI	810	24745		7/24	28.0
1781	0.422	1.675	<0.001	4.0 (+)	ACYPI001342 protein^2^	*Acyrthosiphon pisum*	C4WRW5_ACYPI	355	19792		3/11	18.4
**Carbohydrate processing and metabolism**												
734	1.526	0.7499	0.004	2.0 (−)	polysaccharide deacetylase^1^	*Fusobacterium nucleatum*	gi|495968271	66	69834	9.1	15/62	24
1308	0.732	1.572	0.003	2.1 (+)	ACYPI002851 protein^2^	*Acyrthosiphon pisum*	C4WTT0_ACYPI	818	39779		8/23	28.9
1744	1.601	0.723	<0.001	2.2 (−)	putative diacetyl/L-xylulose reductase^2^	*Acyrthosiphon pisum*	Q201Y9_ACYPI	207	26334		2/17	10.4
1787	0.557	2.395	<0.001	4.3 (+)	ACYPI000057 protein^2^	*Acyrthosiphon pisum*	Q1ZZP5_ACYPI	188	25816		4/18	21.2
**Amino acid synthesis and metabolism**												
913	0.495	1.718	<0.001	3.5 (+)	glutamate dehydrogenase^2^	*Acyrthosiphon pisum*	J9KB74_ACYPI	285	59967		9/44	20.7
975	1.527	0.669	0.005	2.3 (−)	glutamyl-tRNA synthase^1^	*Kyrpidia tusciae*	gi|502839225	78	55629	6.1	14/76	31
1215	0.705	1.690	0.007	2.4 (+)	glutamine synthetase^2^	*Acyrthosiphon pisum*	J9JML2_ACYPI	1783	41935		7/25	22.2
2302	0.615	1.289	<0.001	2.1 (+)	aspartate kinase^1^	*Nitrolancea hollandica*	gi|495756139	58	8291	6.6	4/29	38
**Ion transport**												
1607	0.400	1.895	<0.001	4.7 (+)	ACYPI006090 protein^2^	*Acyrthosiphon pisum*	C4WY80_ACYPI	40	26228		2/21	11.9
1872	1.689	0.802	<0.001	2.1 (−)	ATP synthase oligomycin sensitivity conferral protein^2^	*Toxoptera citricida*	Q5XUB9_TOXCI	157	22719		2/20	9.6
2293	0.457	1.703	<0.001	3.7 (+)	V-type proton ATPase subunit F^2^	*Acyrthosiphon pisum*	Q201X8_ACYPI	1251	13663		5/13	39.3
**Processing and degradation**												
388	1.812	0.546	<0.001	3.3 (−)	transitional endoplasmic reticulum ATPase TER94^1^	*Acyrthosiphon pisum*	gi|193617621	258	89914	5.1	26/36	32
389	1.753	0.748	0.001	2.3 (−)	transitional endoplasmic reticulum ATPase TER94^1^	*Acyrthosiphon pisum*	gi|193617621	187	89914	5.1	27/68	32
1704	0.626	1.325	<0.001	2.1 (+)	proteasome subunit alpha type^2^	*Acyrthosiphon pisum*	C4WUA6_ACYPI	57	27982		2/22	8.3
**Antioxidant**												
1890	0.374	1.700	<0.001	4.5 (+)	uncharacterized protein^2^	*Acyrthosiphon pisum*	J9JUC5_ACYPI	205	27120		2/21	10.5
1908	0.694	1.549	<0.001	2.2 (+)	ACYPI002506 protein^2^	*Acyrthosiphon pisum*	C4WSM1_ACYPI	2239	21794		9/15	48.7
**Biosynthesis of bactericides**												
934	1.328	0.395	0.002	3.4 (−)	radical SAM additional 4Fe4S-binding domain protein^1^	*Acholeplasma sp*.	gi|547179168	73	43424	6.1	8/20	18
**Unknown**												
729	1.757	0.526	<0.001	3.3 (−)	uncharacterized protein^2^	*Acyrthosiphon pisum*	J9JTV1_ACYPI	379	63934		8/29	12.2
731	1.716	0.662	0.002	2.6 (−)	uncharacterized protein DDB_G0274915-like^1^	*Acyrthosiphon pisum*	gi|193716064	91	67060	7.7	12/61	16

^*^Protein abundance pattern in response to *Spiroplasma* infection. (+) = up-regulated protein, (−) = down-regulated protein.

**Number indicates the mass spectrometer used in the spot identification, 1 = Ultraflex II MALDI and 2 = *Synapt* G2 HDMS.

Proteomic analysis of *A. citricidus* using the Ultraflex II MALDI Mass Spectrometer and the *Synapt* G2 HDMS systems rendered the identification of several putative proteins, which are associated with *stress response*, *energy metabolism*, *structural protein* and other functional categories (Tables 1 and 2). Several proteins identified in *A. citricidus* were present as proteoforms. In aphid samples from orange jasmine, we identified two differentially expressed isoforms of the transitional endoplasmic reticulum ATPase-TER94 (spots 388 and 389), six of the repetitive proline-rich cell wall protein 2-like isoform X2 (spots 581, 587, 588, 589, 590 and 660), two of the heat shock protein cognate 3 precursor (spots 663 and 699b), two of the uncharacterized protein J9JJY7 (spots 687 and 693), five of the 60 kDa chaperone (spots 776, 832, 841, 855 and 937), and two for the ATP synthase subunit alpha (spots 959 and 1019) (Table 2). The overall number of differentially expressed proteins in the Ac-BS as compared to the Ac-B sister isoline was much reduced in sweet orange as a host plant if compared to orange jasmine. In this case, only two proteoforms each of the uncharacterized protein J9JWP2 (spots 193 and 194) and the uncharacterized protein J9JJY7 (spots 215 and 217) were found (Table 1). We also had two spots rendering more than one protein hit each. Two different heat shock proteins were identified from spot 699 from aphids reared on orange jasmine (Table 2), and a heat shock protein (262a) and an unchacterized protein (262b) were obtained from mass data analysis of spot 262 from aphids reared on sweet orange (Table 1).

The overall analysis of the differently abundant proteins indicated that *Spiroplasma* regulated the levels of more proteins from the aphid host than from *B. aphidicola* (Tables 1 and 2). However, the proteomic profile obtained from aphids from the two host plants was quite different, indicating that *Spiroplasma* affected *A. citricidus* proteomics differently depending on the host plant.

**Figure 2:**
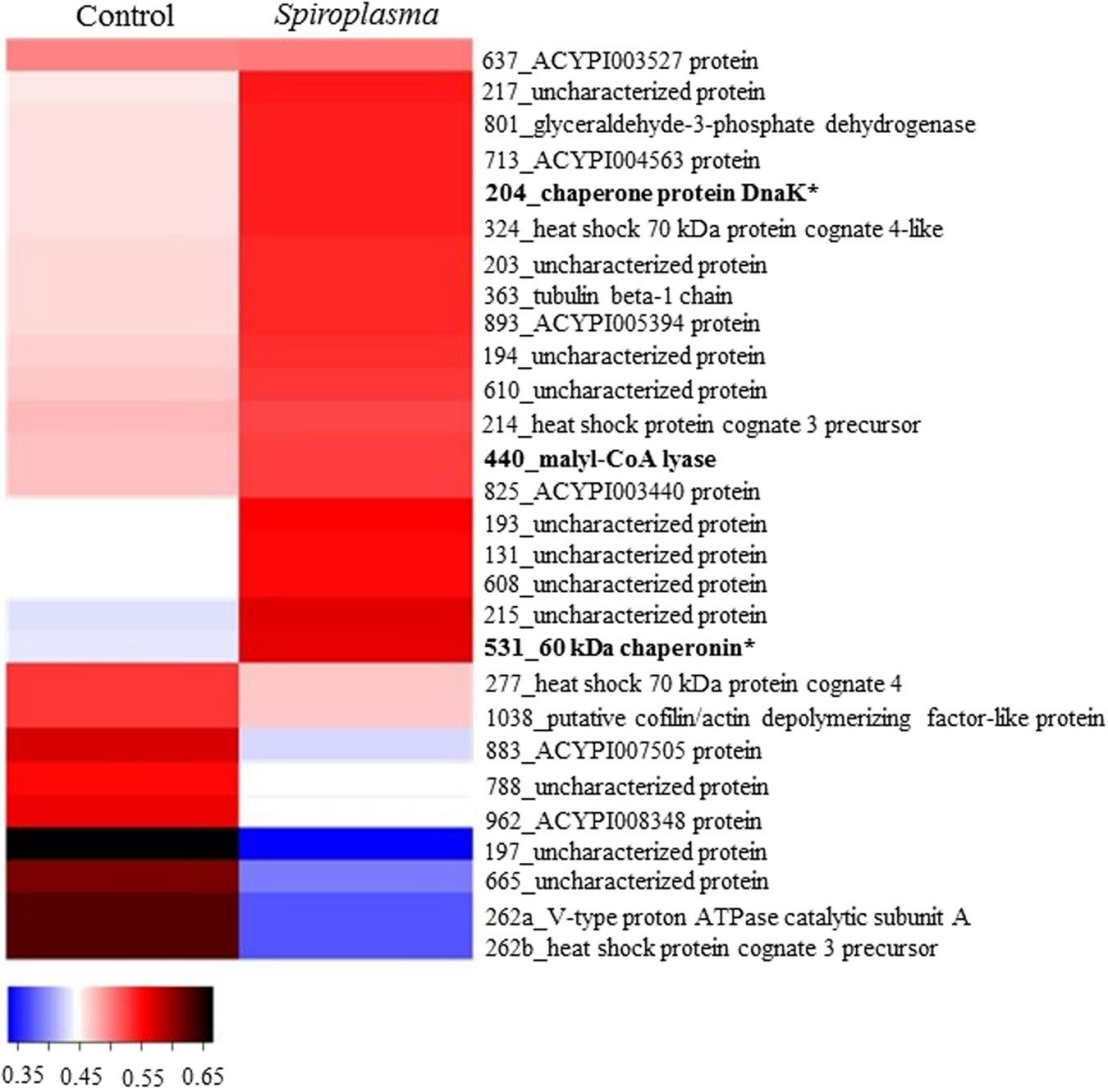
Heatmap of identified proteins on orange jasmine. Proteins are identified by their spot number followed by Blast2Go or Uniprot identification. Regular letters represents proteins from eukaryote, bold letters represents proteins from prokaryote origin, and bold letters with an asteristic (*) represents proteins from *B. aphidicola aphidicola*. Fold change was transformed in a proportional scale in which blue represents the smallest fold change and black represent the largest fold change.

Changes in the proteome of *A. citricidus* induced by *Spiroplasma* infection was less evident in aphids reared in sweet orange, with 19 up-regulated and 9 down-regulated proteins belonging to *protein-protein interaction*, *energy metabolism*, *cell function and structure*, *ion transport* and *processing and degradation* functional groups (Table 1 and Fig. 2). Most of the proteins in aphids reared on sweet orange were uncharacterized, but analysis were made based on their domain. Up and down-regulated proteins were from several functional groups. Most of the eukaryote heat shock proteins and chaperonins from *B. aphidicola* were up-regulated (Fig. 2), and two eukaryote heat shock proteins (spots 274 and 262b), three proteins from *cell function and structure*, one protein from *ion transport*, one from *energy metabolism* and one from unknown functional group were down-regulated. Moreover, down-regulated proteins were all from eukaryote origin, while up-regulated proteins were from both eukaryote and prokaryote (Fig. 2).

**Figure 3:**
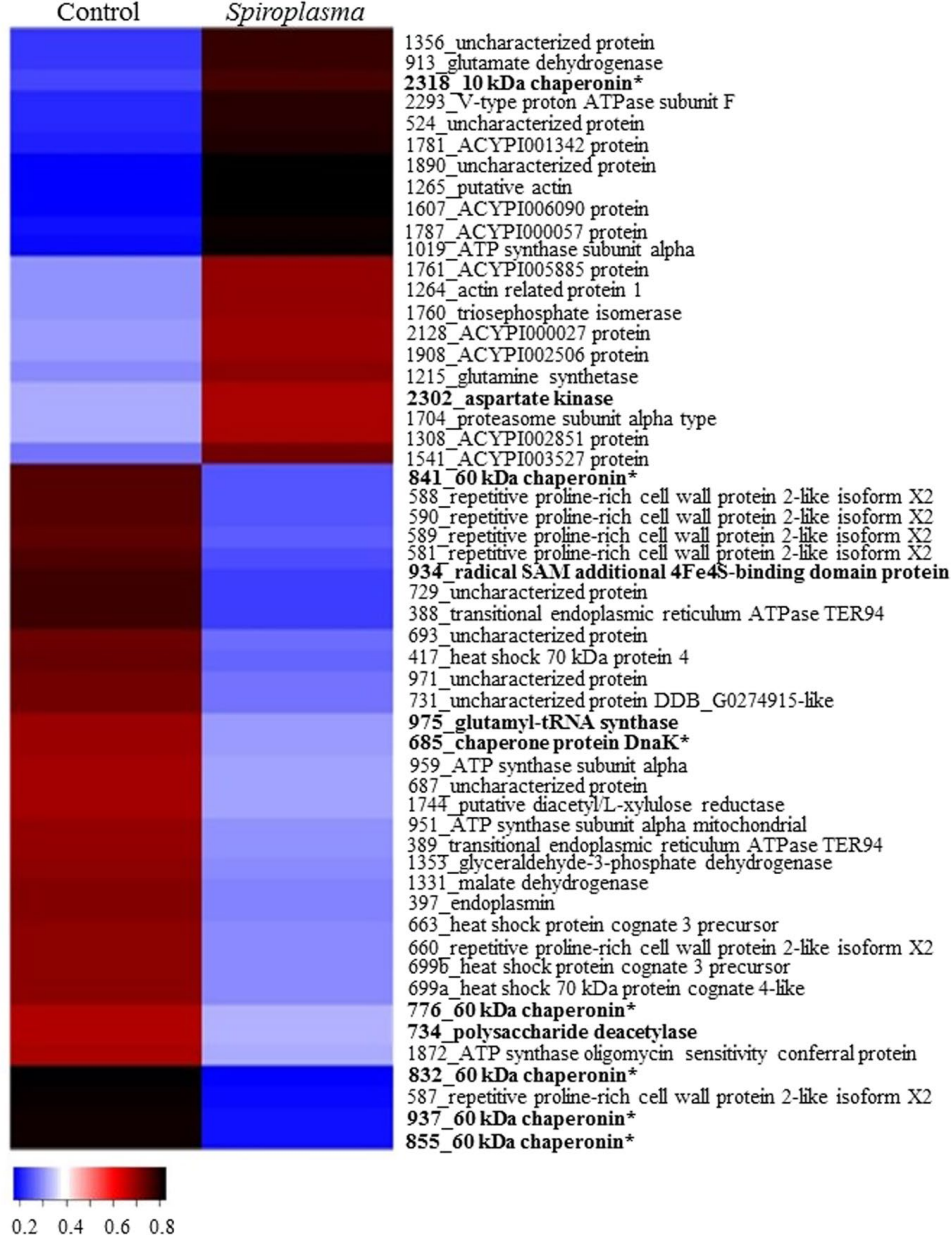
Heatmap of identified proteins on sweet orange. Proteins are identified by their spot number followed by Blast2Go or Uniprot identification. Regular letters represents proteins from eukaryote, bold letters represents proteins from prokaryote origin, and bold letters with asteristic (*) represents protein from *B. aphidicola aphidicola*. Fold change was transformed in a proportional scale in which blue represents the smallest fold change and black represent the largest fold change.

But *Spiroplasma* infection of *A. citricidus* (Ac-BS) feeding on orange jasmine yielded broader changes in the host proteins. Differences in abundance of *antimicrobial peptides*, *carbohydrate processing and metabolism*, *amino acid synthesis and metabolism*, *anti-oxidants* and *biosynthesis of bactericides* was observed only when comparing infected and non-infected aphids fed on orange jasmine. *Spiroplasma* induced the up-regulation of 21 and the down-regulation of 33 proteins (Fig. 3) in orange jasmine-fed aphids. All of the differently abundant proteins of *cell function and structure* and *anti-oxidants*, most of the *amino acid synthesis and metabolism* (except spot 975) and *ion transport* (except spot 1872), and several proteins belonging to other functional groups were up-regulated in *Spiroplasma*-infected hosts (Fig. 3). Down-regulated proteins included mainly those in *protein-protein interaction* and *antimicrobial peptides* functional groups, with three 60 kDa chaperonins (spots 832, 855 and 937) and one repetitive proline-rich cell wall protein 2-like isoform X2 (spot 587) being strongly down-regulated (Fig. 3). The functional groups *energy metabolism*, *carbohydrate processing and metabolism*, *processing and degradation* and *biosynthesis of bactericides* also included proteins that were either up or down-regulated in *Spiroplasma*-infected aphids in orange jasmine. Futhermore, we detected no pattern of protein regulation in regards to protein origin, if eukaryote or prokaryote (Fig. 3).

**Figure 4:**
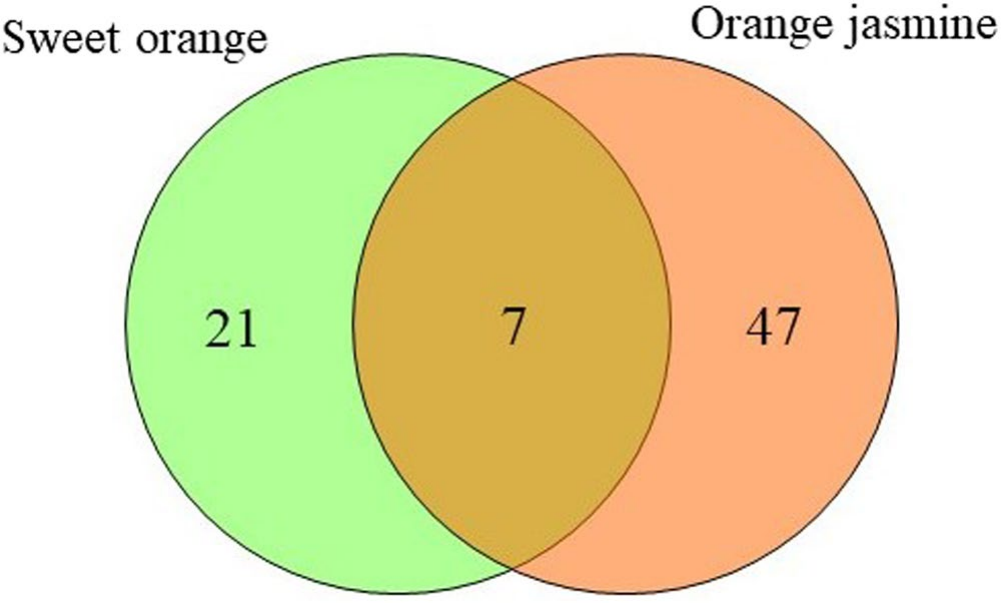
Venn diagram of differently abundant protein spots in sweet orange and orange jasmine.

A comparison of the proteome of *Spiroplasma-*infected *A. citricidus* when exploiting sweet orange and orange jasmine indicated a number of different abundant proteins common to aphids reared on both host plants (Fig. 4), as the uncharacterized protein J9JP34 (spot 524 in orange jasmine and spot 131 in sweet orange), chaperone protein DnaK (spot 685 in orange jasmine and 204 in sweet orange), uncharacterized protein J9JJY7 (spots 687 and 693 in orange jasmine and spots 215 and 217 in sweet orange), 60 kDa chaperonin (spots 776, 832, 841, 855 and 937 in orange jasmine and spot 531 in sweet orange) and ACYPI003527 protein (spot 1541 in orange jasmine and spot 637 in sweet orange) (Tables 1 and 2). But although sharing similar differently abundant proteins in two different host plants, only the uncharacterized protein J9JP34 and ACYPI003527 protein followed the same pattern of abundance in both plants (up-regulation), with the remaining proteins being up-regulated in sweet orange but down regulated in *Spiroplasma-*infected aphids feeding on orange jasmine (Figs 2 and 3). Differently abundant proteins common to both host plants, but with different patterns of expression were represented by heat shock proteins (*protein-protein interaction*) and the ACYPI003527 protein (*cell function and structure*).

## Discussion

Comparative proteomic analysis between *Spiroplasma*-free (Ac-B) and *Spiroplasma-*infected (Ac-BS) sister isolines of *A. citricidus* on two host plants indicated *Spiroplasma* infection affects the host proteome, especially the abundance of proteins belonging to *protein-protein interaction, cell function and energy metabolism* categories. Changes in host proteome due to *Spiroplasma* infection were quite different in aphids feeding on different host plants - orange jasmine or sweet orange, suggesting that some of the differences observed between host plants for infected or uninfected aphids might be due to the interaction of the host plant and the infection status of the aphid. Due to a number of issues with aphids survival after microinjection and consequently the amount of protein obtained, we could not perform gels that would allow comparing differences in proteins abundances according to the host plant for a given infection status. Our data allows only comparisons between infection status for a given host plant. The huge differences in the aphid proteomics when feeding on sweet orange and orange jasmine certainly require additional studies to clarify the role of the host plants on the proteomics of *A. citricidus*.

One of the major groups of proteins affected by *Spiroplasma* infection, regardeless the host plant was the heat shock proteins (HSPs). HSPs are ubiquitous proteins common to prokaryotes and eukaryotes and are expressed either under stress or no-stress conditions^34^. HSPs are divided into families based on their molecular weight and sequence homology^34,35^. Function of HSPs proteins belonging to different families may depend on the development stage, subcellular location, and environmental conditions^36^. Therefore, the role of HSPs depend on other proteins they may interact with, which changes depending on the physiological state of the aphid in a given moment.

*Spiroplasma* affected the expression of several HSPs (HSP10, HSP60, HSP70 and HSP90) from *A. citricidus* and *B. aphidicola*. All of the identified HSP10 and HSP60 and one HSP70 (DnaK) were similar to those from *B. aphidicola*, and the remaining HSP70 and HSP90 were similar to those from *A. pisum*. But the outcome of the regulation of HSPs expression by *Spiroplasma* was quite different depending on the host plant the aphid was developing on. While the majority of the differently abundant HSPs were up-regulated in aphids developing on sweet orange, HSPs of aphids on orange jasmine were down-regulated.

The down-regulation of the *B. aphidicola* HSPs, GroEL (HSP60) and DnaK (HSP70) in infected aphids in a less suitable host (orange jasmine) demonstrate that *Spiroplasma* + orange jasmine can negatively interfere, directly or indirectly, with the exchange of molecules between the primary symbiont *B. aphidicola* and the aphid, even though a GroES was up-regulated. The regulation of different groups of HSPs demonstrate that the interaction between the aphid and *B. aphidicola* metabolism can differ depending on the changes induced by *Spiroplasma* in aphids feeding on food sources with different nutritional quality.

Two other abundant groups of proteins, *energy metabolism* and *cell function and structure*, were differentially expressed in response to *Spiroplasma* infection, regardless the host plant the aphid was feeding on. HSPs activity are closely linked to ATP availability^35,36^. Thus, down-regulation of HSPs in aphids feeding on orange jasmine were followed by down regulation of most of the ATP synthases produced, but no such correlation was observed in infected aphids feeding on sweet orange. Additionally, HSP are also reported to interact with cytoskeletal proteins^37^. We did observe up-regulation of proteins included in *cell function and structure*, including putative actins and actin-related proteins. But over-expression of these proteins in *Spiroplasma*-infected aphids was observed on both host plants, even under conditions in which HSP expression was reduced. Therefore, cell function on both host plant interacts with *Spiroplasma* in the same way to affect aphid’s actin proteins.

HSPs are also reported to play a role in immunity by interacting with antimicrobial peptides^38,39^. Proline-rich peptides are common antimicrobial peptides reported to produce pores in Gram^-^ bacteria^40^ and play key roles in the immune response against microbials through interference with protein folding and protein expression^41–43^. A strong down-regulation of HSP70 and antimicrobial peptides was observed in *Spiroplasma*-infected aphids developing on orange jasmine. Differentially expressed *antimicrobial peptides* were all isoforms of repetitive proline-rich cell wall protein 2-like isoform X2. Down-regulation of the host immune system in *Spiroplasma*-infected aphids was also observed by the down-regulation of SAM binding domain–containing proteins, which are linked to various bacteriocin biosynthesis proteins^44^.

Aphids are known to use a nutrition-based mechanism to control amino acid provisioning to regulate the growth of *B. aphidicola*^45^. Abundance of DnaK of *B. aphidicola* reflected the host plant suitability. DnaK abundance was higher in infected aphids feeding on sweet orange while in orange jasmine DnaK abundance was down-regulated in infected aphids.

Thus, it seems that *Spiroplasma* can affect the immune system of the host and/or the nutritional mechanism that regulates *B. aphidicola* growth. Glutamine is one of such nutrients provided by the host^3,45^. In orange jasmine, we observed an increase in glutamine synthase and glutamate dehydrogenase proteins, which are enzymes involved in the recycling of ammonia and synthesis of glutamine and glutamate. Both are required for the synthesis of amino acids and purine metabolism in *B. aphidicola*-carrying bacteriocytes^45^. Regulation of these amino acids has been proposed as key in maintaining amino acids availability balanced for both the host and the symbiont^45^. However, we did not detect up-regulation of glutamine transporters in the protein spots we were able to analyze to demonstrate enhanced transport of glutamine from the hemolymph to the bacteriocyte^46,47^.

Aspartate is also a non-essential amino acid provided by the host that has to be transported into *B. aphidicola* cells. Aspartate is an important source of nitrogen for the synthesis of essential amino acids^45^. Additionally, we observed an increase in a prokaryote aspartate kinase, an enzyme involved in the synthesis of essential amino acids (lysine and threonine) through aspartate phosphorylation^45,48^. Up-regulation of aspartate kinase is suggestive of increased synthesis of essential amino acids from aspartate, but malate dehydrogenase was down-regulated in *Spiroplasma*-infected aphids in orange jasmine. Malate dehydrogenase is the enzyme involved in the transamination of oxaloacetate to produce aspartate, but in aphid bacteriocytes synthesis of aspartate is based on the precursor glutamine. However, down-regulation of the aphid malate dehydrogenase enzyme may be correlated with the observed reduction in aphid energy metabolism, as oxaloacetate is also an intermediate of the citric acid cycle.

Moreover, up-regulation of *antioxidants* and proteasome subunit alpha type proteins in *Spiroplasma-*infected aphids feeding on orange jasmine may also indicate increased cell damage and the requirement for cell protection and degradation of unneeded or degraded proteins. Antioxidants protects cells by reducing the negative effects of the accumulation of reactive oxygen species^49^, while proteasomes degrade damaged and misfolded proteins^50,51^. Proteasome subunit alpha type proteins belong to a selective proteolytic protein machinery (proteasome) acting on ubiquitin-tagged proteins, even though proteins from the ubiquitinylation pathway were not identified among the analyzed proteins. Up-regulation of proteasome subunit alpha type proteins indicates the increased need of protein degradation, as proteasome deficiency could lead to the accumulation of damaged and misfolded proteins and result in a number of pathologies^50,51^. The requirement of high levels of proteasome activity may also be associated with the reduced availability of HSPs, which in turn may have led to an accumulation of damaged and/or misfolded proteins.

In conclusion, *Spiroplasma* infection affects the aphid-primary symbiont proteome mainly by regulating the activity of heat shock proteins and other processes that HSPs participate, such as energy metabolism and immune system. However, the effects of *Spiroplasma* infection on the host aphid proteomics differed between host plants, suggesting that some of the differences could be a result of the interaction between the infection status of the host and the nutritional quality of the host plant. We propose that *Spiroplasma*-infected aphids feeding on orange jasmine invest to provide a better nutritional environment to its primary symbiont, *B. aphidicola* and reduce any fitness cost associated with the *Spiroplasma* infection under such nutritional stress condition in a controlled trade-off between immune system regulation and nutritional deficiency.

Further studies to investigate the fitness costs of *A. citricidus* association with *Spiroplasma* in response to immune challenges under different nutritional stress conditions and the impact of nutrition alone will help to shed light on the role this secondary symbiont may play in aphid biology and on the costs involved in such association.

## Material and Methods

### Insect isolines

Sister isolines of *A. citricidus* infected (Ac-BS) or not (Ac-B) with *Spiroplasma* were established as described elsewhere^33^. Briefly, part of an isoline of *A. citricidus* free of secondary symbionts was microinjected with hemolymph from *Spiroplasma*-infected *A. aurantii*, while the other part was microinjected with PBS buffer (137 mM NaCl, 2.7 mM KCl, 10 mM Na_2_HPO_4_, 2 mM KH_2_PO_4_). Thus, infected and control lines of *A. citricidus* had the same genetic background.

Ac-B and Ac-BS sister lines were each maintained on seedlings of sweet orange (*Citrus sinensis* var. Pera) and orange jasmine (*Murraya paniculata*) inside rearing cages (50 cm high × 15 cm diameter), containing two lateral openings closed with cloth for ventilation, and maintained under controlled condition (25 ± 2 °C; 60 ± 10% UR; photoperiod 14 L:10D h).

### Protein extraction

Adults from Ac-B and Ac-BS were collected from their host plants and subjected to protein extraction. Three biological replicates of two-hundred and fifty aphids each per host plant and isoline were crushed in 1 mL of extraction buffer (8 M Urea, 4% DTT, 4% CHAPS) and incubated in ice for 40 min. The material was centrifuged (12,000 g × 15 min × 4 °C) and the supernatant was transferred to a new tube for purification with the commercial system 2D - Clean up (GE Healthcare®). Briefly, purification was made by adding 300 μL of the precipitant buffer followed by incubation on ice for 15 min. Afterwards, 300 μL of co-precipitant was added and the sample centrifuged (12,000 g × 5 min × 4 °C) once again. The supernatant was discarded and 40 μL of co-precipitant was layered on top of the pellet. The pellet was allowed to sit on ice for 5 min. The sample was centrifuged (12,000 g × 5 min × 4 °C) and the pellet recovered in 25 μL of Milli-Q, 1 mL of wash buffer and 5 μL of wash additive. The purification procedure was finalized after centrifugation (12,000 g × 5 min × 4 °C) and resuspension of the pellet in UT buffer (7 M Urea, 2 M Thiourea, 20 mM Tris, 2% CHAPS). The pH was measured using Neutralit pH 5.5–9 strips (Merck) and adjusted to pH 8.5 with 1 M NaOH. Total protein concentration was verified by using the commercial system RC DC Protein Assay (Bio-Rad^®^), following the manufacturer instructions.

### 2D-gel electrophoresis

Proteomic analyses were made separately for protein samples collected from aphids from different host plants; hence, the effect of *Spiroplasma* infection on the aphid proteome was individually analyzed within each host plant. For each biological replication (3) four technical replication were performed; thus a total of 12 images were analyzed per host plant for each status of infection.

To perform analytic 2D gels, protein extracts were labeled with one of the three CyeDye (GE healthcare) in a total of 25 µg of protein followed by first dimension protein separation by electrofocalisation on IPG strip (pH3-10NL, 24 cm, GE Healthcare), following the manufacturer instructions. Therefore, each Ac-B and Ac-BS sample from both experimental host plants was labeled in duplicates with Cye3 and Cye5 leading to a total of 4 replicates per treatment. All IPG strips also carried the internal standard labeled with Cye2. The internal standard was composed of an equimolar mixture of Ac-B and Ac-BS.

The solution with labeled proteins was adjusted to a final volume of 450 µL of UT buffer, 2 M dithiothreitol (DTT) and 0.8% v/v IPG ampholyte (SERVA). IPG strips were set on a PROTEAN i12 IEF (Bio-Rad) for active rehydration for 11 h at 50 V, followed by isoelectric focalization (IEF) at 200 V for 200 Vh, 500 V for 500 Vh, 100 V for 100 Vh, and 8000 V for 60000 Vh. Both steps were conducted at 15 °C and 50 µA maximum/IPG strip.

After IEF, IPG strips were prepared for second dimension electrophoresis. First, IPG strips were equilibrated for 15 min in 375 mM Tris (pH 8.8), 6 M Urea, 20% glycerol (v/v), 2% SDS and 130 mM dithiothreitol (DTT), followed by an extra 15 min in a similar buffer, in which DTT was replaced by 135 mM iodoacetamide (IAA).

Once treated, IPG strips were positioned onto a 2D 12.5% HPE large gel (SERVA^®^). Second dimension electrophoresis used the HPE FlatTop tower (GelCompany) under the following conditions: phase I − 100 V, 7 mA, 1 W for 30 min, 200 V, 13 mA, 3 W for 30 min, and 300 V, 40 mA, 5 W for 10 min; phase II − 1500 V, 40 mA, 30 W for 3 h 50 min, and 1500 V, 45 mA, 40 W for 50 min. IPG strips were removed after phase I, and only 2D gel was used in phase II.

### Image analysis and spot selection

2D gel images were captured with Typhoon Fluorescence Imager (Amersham) at wavelengths corresponding to each dye. A total of 12 images were analyzed with the software Progenesis SameSpots (Nonlinear Dynamics). Statistical analysis of protein expression levels were determined for each spot based on mean spot volume (n = 12), and differences in protein expression between control and *Spiroplasma*-infected aphids were detected using ANOVA. Spots with a p ≤ 0.05 were selected for inclusion in the results. In the experiment with orange jasmine as a host plant, only spots with a significant *p* value and with a change in ratio higher than two (fold change ≥ 2) were selected, once spots with a fold change lower than 2 were considered dubious.

After the list of spots was defined, a preparative gel was made, and another IPG strip loaded with internal standard labeled with Cye2 and 500 µg of unlabeled proteins (250 µg Ac-B and 250 µg Ac-BS) was subjected to electrofocusing. This IPG strip was later used for a second dimension electrophoresis as earlier mentioned. The image of this preparative gel was aligned with the previously obtained gels to locate the spots of interest. Spot picking was made in duplicate using an Ettan Spot Picker Robot (GE Healthcare).

### Mass spectrometry using an Ultraflex II MALDI Mass Spectrometer

Excised spots were designated for the Proteineer dp automated Digester (Bruker, Bremen, Germany). Briefly, gel pieces were washed with three incubations in 100% of 50 mM ammonium bicarbonate, followed by incubations in a 1:1 mixture of acetonitrile (ACN) and 50 mM ammonium bicarbonate. Two additional washes were performed with 100% acetonitrile to dehydrate the gel. Gel pieces were first soaked in freshly activated trypsin (Porcine, Proteomics Grade, Roche) at 8 °C for 30 min, and later subjected to protein trypsinization for 3 h at 30 °C. Peptide extractions were performed with 10 µL of 1% formic acid (FA) for 30 min at 20 °C.

Protein digests (3 µL) were adsorbed for 3 min on pre-spotted Anchorchips (R) using the Proteineer dp automaton. Spots were washed on-target using 10 mM ammonium dihydrogen phosphate in 0.1% trifluoroacetic acid (TFA) and desalted after washing with MilliQ water (Millipore). High throughput spectra were acquired using an Ultraflex II MALDI Mass Spectrometer (Bruker) in positive reflectron mode with close calibration enabled.

Successful spectra were summed, treated, and de-isotoped in line with an automated SNAP algorithm using Flex Analysis 2.4 software (Bruker), and subsequently submitted in batch mode to the Biotools 3.0 software suite (Bruker) with an in-house hosted MASCOT search engine (www.MatrixScience.com). Three databases were used: (1) the public National Center for Biotechnology Information (NCBI) non-redundant database, (2) the public National Center for Biotechnology Information (NCBI) non-redundant database with parameters set for Arthropoda, and (3) a homemade database containing all available aphid and aphid-symbiont protein sequences. A mass tolerance of 100 ppm with close calibration and one missing cleavage site were allowed. Partial oxidation of methionine residues and complete carbamylation of cystein residues were considered. The probability score calculated by the software was used as a criterion for correct putative identification.

In order to confirm identifications, experimental molecular weight (MW) and pI were compared to the predicted values resulting from the MASCOT analysis.

### Mass spectrometry using *Synapt* G2 HDMS

Another range of excised spots were dehydrated with 100% ACN, followed by reduction and alkylation. Reduction was performed in 20 mM DTT and 50 mM ammonium bicarbonate for 40 min at 55 °C. The supernatant was removed and alkylation performed in 55 mM IAA and 50 mM ammonium bicarbonate for 30 min in the dark at room temperature. The supernatant was removed and gel fragments were washed in 25 mM ammonium bicarbonate.

Gel fragments were dehydrated with 100% ACN and rehydrated with 20 ng/µL trypsin (Promega V5111) in 50 mM ammonium bicarbonate. Protein digestion with trypsin was carried out at 37 °C for 16 h; after this period, enzymatic activity was blocked by a solution of 50% ACN and 5% FA in ultrapure water. Peptides were first washed in 60% methanol (MeOH) and 1% FA in ultrapure water, followed by a wash in 50% ACN and 1% FA in ultrapure water, with a final wash in 100% ACN. In each wash, peptides were sonicated for 20 min at 45 °C (Ultrassom, Thronton). The supernatant was transferred to a new vial, and dried at room temperature in a vacuum centrifuge (Eppendorf 5301).

Dried samples were rehydrated in 10 µL 0.1% TFA in water and purified using a reverse phase column (Reverse phase Zip-Tip C18, Millipore) following manufactorer’s instructions. Samples were eluted in 15 µL of 0.1% TFA acidified water and 8 µL were injected in a NanoAcquity UPLC (Waters, Manchester, UK) connected to a *Synapt* G2 HDMS. Peptides were passed through a C18 Symmetry capture column (5 μm, 180 μm × 20 mm) and separated on a silica-based, reverse phase C18 HSS T3 column (1.8 μm, 75 μm × 100 mm) (Waters, Manchester, UK). Mobile phase was composed of solution A (100% H_2_O + 0.1% FA) and solution B (100% ACN + 0.1% FA) starting with a gradient of 99% A and 1% B for 90 min, and then 60% A and 40% B for 2 min and 15% A and 85% B for 3 min. The original condition of the column was reestablished for 25 min, totalling 120 min/run at 300 µL/min.

MS data acquisition used a Q-TOF in the *Synapt* G2 HDMS equipped with an ion mobility cell and a nano lock spray source in the positive ion and ‘V’ mode. The human (Glu1)-fibrin-peptide (GFP) (1 *p*mol.μL^−1^) was used to calibrate the *Synapt* G2 HDMS with data post-acquisition lock mass using the GFP double charged precursor ion, with sampling of the reference spray at a frequency of 30 s. MS experiments were performed by switching between low (3 eV) and high collision energies (15–50 eV) applied to the T-wave cell trap filled with argon. The low and high energy scans from *m/z* 50 to 2000 used a scan time of 0.8 s.

Analysis of MS data was performed using ProteinLynx GlobalServer version 3.1 (Waters) with reverse Uniprot databases: *Acyrtosiphum pisum*, *Myzus persicae*, *Toxoptera* sp., *B. aphidicola* and *Spiroplasma* sp., accessed in August 14th, 2015. Uniprot database of aphids included their secondary symbionts. Analysis followed parameters automatic tolerance for precursor and ion product, minimum of three consecutive fragment-ions for peptide, minimum of seven consecutive fragments-ions for protein, minimum of two peptides for protein, and only one missed cleavage for trypsin, carbamylation of cysteine with fixed modification and methionine oxidation with variable modification; and false discovery rate (FDR) maximum of 4%.

Spots that yield good spectra in both methods adopted not always rendered identical putative protein identification due to differences in databases. Spots that rendered diverse hits on both mass spectrometry systems were assigned to the best hit by considering coverage (%), expected mass in both analysis, and the estimated error (ppm) obtained with the Ultraflex II-MALDI System.

All identified proteins were classified into functional groups. Groups were set based on ontology, protein domain and homology after similarity searches in the Uniprot, String, InterPro and Pfam databases.
